# Weaknesses in Experimental Design and Reporting Decrease the Likelihood of Reproducibility and Generalization of Recent Cardiovascular Research

**DOI:** 10.7759/cureus.21086

**Published:** 2022-01-10

**Authors:** John L Williams, Hsini (Cindy) Chu, Marissa K Lown, Joseph Daniel, Renate D Meckl, Darshit Patel, Radwa Ibrahim

**Affiliations:** 1 College of Osteopathic Medicine, University of New England, Biddeford, USA

**Keywords:** checklist, bias, statistical power, power, arrive, preclinical research, cardiovascular research, irreproducibility, reproducibility

## Abstract

Recent evidence indicates that many clinical and preclinical studies are not reproducible. Prominent causes include design and implementation issues, low statistical power, unintentional bias, and incomplete reporting in the published literature. The primary goal of this study was to assess the quality of published research in three prominent cardiovascular research journals by examining statistical power and assessing the adherence to augmented ARRIVE guidelines (Animal Research: Reporting of In Vivo Experiments). For unpaired t*-*tests, the average median power for a 20% and 50% change was 0.27 ± 0.06 and 0.88 ± 0.08, respectively. For analysis of guidelines, 40 categories were assessed with a 0-2 scale. Although many strengths were observed, several key elements that were needed for reproducibility were inadequate, including differentiation of primary and secondary outcomes, power calculations for group size, allocation methods, use of randomization and blinding, checks for normality, reports of attrition, and adverse events of subjects, and assessment of bias. A secondary goal was to examine whether a required checklist improved the quality of reporting; those results indicated that a checklist improved compliance and quality of reporting, but adequacy levels in key categories were still too low. Overall, the findings of this study indicated that the probability for reproducibility of many clinical and preclinical cardiovascular research studies was low because of incomplete reporting, low statistical power, and lack of research practices that decrease experimental bias. Expansion of group sizes to increase power, use of detailed checklists, and closer monitoring for checklist adherence by editors and journals should remediate many of these deficits and increase the likelihood of reproducibility.

## Introduction

Reproducibility of research findings and translation of preclinical research to potential clinical applications are current issues of considerable concern [1]. A variety of factors may account for the discrepancies in the findings between similar research studies, including undetected differences in the design, protocols, and conduct of the studies; variations in the nature of subjects and reagents; inconsistent methods for the analysis of data; and weak reporting of study results [2,3]. Low statistical power, inappropriate use of statistics, pseudoreplication, and poor control of experimental bias with procedures such as randomization and blinding have emerged as major problems [4,5].

To address these shortcomings, guidelines for the design and reporting of clinical or preclinical research have been widely supported by journals, academic institutions, and private and governmental funding agencies. For preclinical research, the ARRIVE guidelines (Animal Research: Reporting of In Vivo Experiments) have been embraced by over 1,000 research publications [6]. ARRIVE and other guidelines that have been adopted cover a full spectrum of items that should be reported in research publications to increase the rigor of the reports and increase the likelihood that technical aspects of research are better replicated and the true findings can be reported fully.

Surprisingly, the adoption of ARRIVE and other guidelines has only minimally increased the quality of biomedical research, and adherence to the guidelines by researchers has remained low [7-9]. Some publications have recommended or required that investigators use a checklist to better monitor the use of the elements and report the many items that are needed for a strong study design and a complete research report. In some cases, the overall quality or completeness improved, but the use or reporting of many key elements, such as randomization, blinding, attrition of experimental subjects, and power analysis for selection of group size, often remained unacceptably low [8,10]. It appears that recommendations alone for the use of a checklist by journals are not sufficient to assure compliance by authors. More active involvement by reviewers, editors, publishers, academic institutions, and funding agencies is needed so that checklists are required and more fully monitored for compliance. In addition, the use of the Good Laboratory Practice guidelines by research institutions and laboratories may be useful to ensure the integrity and quality of design, conduct, and results of preclinical research studies [11].

The primary objective of this study was to assess whether relevant indicators of reproducibility were adequate in the recent cardiovascular research literature. For this assessment, we used two approaches in three major cardiovascular research journals. First, we calculated the statistical power of unpaired t*-*tests used in the research to detect a meaningful change; second, we rated the performance of the research reports for adherence to ARRIVE and other guidelines. Secondary objectives were (1) to determine whether differences might exist between journal volumes for the ARRIVE scores and (2) to examine the possible impact of using a checklist that contained detailed guidelines on the quality of reporting.

Our findings indicate that there was adequate use and reporting of several indices of reproducibility in the cardiovascular research journals that we examined. In contrast, however, the quality of a number of other important criteria fell far below acceptable levels. Low statistical power was observed, which contributed to high probabilities of false negatives and false positives. With some notable exceptions, there was generally a low prevalence for the reporting of randomization and allocation methods, blinding, attrition, adverse events, and bias. Our results suggest that the use of a detailed checklist for authors upon submission of a manuscript improves the compliance with recommended or required guidelines, but even with the use of a checklist, the compliance with some important categories in the checklist is often far too low. More active monitoring of adherence to the guidelines and checklists by reviewers, editors, and publishers is recommended.

## Materials and methods

In this study, power analysis and assessments of adherence to accepted guidelines were used as indices of reproducibility. Articles were examined from three prominent peer-reviewed cardiovascular research journals: (1) *American Journal of Physiology: Heart and Circulatory Physiology*, (2) *Circulation Research*, and (3) *Cardiovascular Research*. In the text and figures of this paper, these journals are abbreviated as AJP:HC, Circ Research, and CV Research, respectively. The journal volumes and articles that were used for the analyses are summarized in Table 1. The selection of the specific journal volumes and articles was preplanned as described in each section. The journal articles that were included in these analyses are listed in the supplement that is accessible online (https://doi.org/10.6084/m9.figshare.16890544.v1).

**Table 1:** Journal volumes reviewed and the types of analysis conducted. AJP:HC = American Journal of Physiology: Heart and Circulatory Physiology; Circ Research = Circulation Research; CV Research = Cardiovascular Research.

Journal	Volume	Dates	Power analysis	Guidelines analysis
AJP:HC 2017	312	January-June, 2017	X	X
AJP:HC 2019	316	January-June, 2019	X	X
AJP:HC 2020	319	July-December 2020	X	
AJP:HC 2021	320	January-March 2021	X	
Circ Research 2019	124	January-June, 2019	X	X
CV Research 2019	115	January-June, 2019	X	X
CV Research 2020	116	January-June, 2020	X	

Analysis of power

Journal articles with the volumes and dates that are indicated in Table 1 were examined for acceptability for power analysis. The analysis of statistical power focused on the power of unpaired t*-*tests; therefore, articles were excluded if they did not use unpaired t*-*tests. In addition, articles were excluded if the data were not presented in an acceptable form for analysis in the figures or tables. This study examined the statistical power of unpaired t*-*tests in AJP:HC articles in 2017, 2019, 2020, and 2021, Circ Research articles in 2019, and CV Research articles in 2019 and 2020. Because the months of AJP:HC volumes 319 and 320 were continuous (July through December 2020 and January through March 2021, respectively), those datasets were combined for analysis and presentation. Because of financial constraints, some articles in Circ Research that required additional payments beyond the regular university library subscription rate at the time of the analysis were excluded.

In inferential statistics, samples should be selected that fairly represent a larger population. As a result, therefore, the results and conclusions from the sample data can then be inferred to the larger group. The power of an inferential statistical test is the probability of that test to detect an actual difference in the population between the groups [12]. A false-negative test result occurs when a statistical test with samples is negative but an actual difference exists between the entire groups of the populations. In a statistical test, the probability of a false negative (beta) is calculated as 1 minus the probability value of the power. A false-negative result on a statistical test is referred to as a type II error.

In our studies, we examined the prospective (a priori) power and type II error probabilities of unpaired t*-*tests (also referred to as independent samples t*-*tests or two-sample t-tests) in the research articles. Unpaired t-tests were selected for study because they are used frequently by cardiovascular research scientists, and power calculations for these tests usually can be determined easily from the summary data that are presented. For unpaired t-tests, these calculations involve a stated critical P-value (alpha), the number of subjects in each of the two groups (*n*), and estimated effect size (ES) that is meaningful [12]. This ES is determined from the standard deviation (SD) of the groups and the size of the difference between the groups that are considered to be meaningful or important (*d*). For unpaired t*-*tests, ES (often referred to as Cohen’s d) equals the difference between the means divided by SD [12].

In medical sciences, the magnitude of *d* is relative, varying among different areas of investigation. Therefore, for calculations of power of unpaired t*-*tests in this analysis, we did not attempt to determine what effect sizes might be considered small, medium, or large for the many different types of data that were reported in the examined articles. In this analysis, *d* was set as the absolute value of a 20% or 50% difference from the initial mean value (i.e., the control, baseline, or first mean value) that was presented in a table or figure. This type of power calculation was performed both for results that were reported to be significantly different statistically as well as for those that were statistically not significant (Table 2). Power calculations were based on an alpha of 0.05 for a two-tailed t-test.

**Table 2:** Numbers of unpaired t-tests that were analyzed in the articles. Total tests, significant tests, and NS tests refer, respectively, to total numbers of unpaired t*-*tests, those that were reportedly statistically significant, and those that were not statistically significant. AJP:HC = American Journal of Physiology: Heart and Circulatory Physiology; Circ Research = Circulation Research; CV Research = Cardiovascular Research.

Journal	Volume	Total articles	Total tests	Significant tests	NS tests
AJP:HC 2017	312	37	386	216	170
AJP:HC 2019	316	37	491	237	254
AJP:HC 2020	319	33	380	195	185
AJP:HC 2021	320	21	198	111	87
AJP:HC total		128	1,455	759	696
Circ Research 2019	124	26	262	151	111
CV Research 2019	115	37	337	149	188
CV Research 2020	116	40	399	258	141
CV Research total		77	736	407	329
Totals		229	2,453	1,317	1,136

For measurements from a figure, the magnitude of the initial value was obtained by measuring the difference in the pixels of the bar in the graph from the zero value of the graph. These measurements were made on the screen of an Apple Macintosh computer (Apple Inc., Cupertino, CA) using the pixel values of the crosshairs during the screen capture function (shift-command-4). Measurements of either SD or standard error of the mean (SEM) were calculated as the difference in pixels from the value of the measured variable to the SD or SEM bar in the graph. If the lines in the figure were blurry, the values were not measured. For tables, the initial values and the SDs or SEMs for these dependent variables were taken directly from the values in the tables. The unit that is used in this type of measurement is irrelevant because the power calculation normalizes the value of the dependent variable by division of this value by the measured SD. For data expressed as mean and SEM, SD was calculated as the mathematical product of SEM and the square root of *n*. A maximum of 50 baseline or control measurements was taken from any paper.

This study determined the power of unpaired t*-*tests to detect a change of either 20% or 50% from the initial values with a value of 0.05 using either G*Power (Heinrich Heine University Düsseldorf, Düsseldorf, Germany) or R data analysis software (R Foundation for Statistical Computing, Vienna, Austria). The measured SD of the initial group was used for both the initial group and the group with a calculated 20% or 50% change. A total of 2,453 unpaired t*-*tests were analyzed from 229 recent articles in three prominent peer-reviewed cardiovascular journals (Table 2).

Analysis of guidelines

General compliance with the recommendations of the ARRIVE guidelines was assessed in articles that were published in AJP:HC, Circ Research, and CV Research (Table 1). Most of the categories that were used for this analysis (Table 3) were initially derived from the 2010 ARRIVE guidelines [13] that have recently been modified [14]. Two other categories were included that incorporated other important information (Table 3, categories X and GG). Because we lacked scientific expertise in most of the content areas, we did not attempt to interpret the results or determine whether the results addressed the objectives or hypothesis (ARRIVE category 18a) [13].

**Table 3:** Questions used for the analysis of adherence to guidelines and recommendations. Cat refers to reference letters that are used for each category in the text. ^1 ^Original ARRIVE guidelines [13]. ^2^ Revised ARRIVE guidelines [14]. ARRIVE = Animal Research: Reporting of In Vivo Experiments; IACUC = Institutional Animal Care and Use Committee; IRB = Institutional Review Board.

Cat	Descriptor	Section	2010^1^	2020^2^	Question summary
A	Title	Title	1		Was the title as accurate and concise as possible?
B	Abstract	Abstract	2	11	Was the abstract an accurate summary of the background, goals, methods, findings, and conclusions?
C	Background	Introduction	3a	12a	Did the introduction include adequate background of the context and experimental approach?
D	Model	Introduction	3b	12b	Did the introduction explain how the experimental model addresses the experimental objectives?
E	Objectives	Introduction	4	13	Did the introduction clearly describe the objectives or hypothesis of the study?
F	IACUC/IRB	Methods	5	14	Did the methods adequately address whether the study conformed to ethical standards or review?
G	Groups (IV)	Methods	6a	1a	Were the number of experimental and control groups (independent variables) described?
H	Randomization	Methods	6b	4a	Were steps taken to reduce bias through randomization of treatments or measurements?
I	Blinding	Methods	6b	5	Were steps taken to reduce bias through blinding during measurements and analysis of data?
J	Units	Methods	6c	1b	In the methods, were details given on what units were used in the experiments (animals/subjects, tissues, cells, etc.)?
K	How/why	Methods	7a,d	9a,d	In the methods, were procedures described in detail and the rationale given for the methods?
L	When/where	Methods	7b,c	9b,c	Were details given describing details of when (e.g., time of day) and where procedures occurred?
M	Animals	Methods	8	8	Was information given about the species, strain, sex, age, weight, and source of animals used?
N	Housing	Methods	9	15	Was there specific information on the types of facilities and husbandry conditions?
O	Group numbers	Methods	10a	2a	In the methods, was the total number of subjects or units used and the numbers in each experimental group specified?
P	Power	Methods	10b	2b	Was there an explanation of how the number of subjects (or other units) needed was determined?
Q	Allocation	Methods	11a	4b	Were details given on allocation to treatment groups (e.g., randomization or matching)?
R	Treatment order	Methods	11b	4b	Was information detailed on the order of treatments or assessment of experimental subjects (or units) within the groups?
S	Outcomes	Methods	12	6a	In the methods, were the experimental outcomes that were assessed clearly defined?
T	1°/2° outcomes	Methods	12	6b	Were the experimental outcomes that were assessed delineated as primary and secondary outcomes?
U	Statistics	Methods	13a,b	7a	Were the statistics that were used described clearly in detail, including for the analysis for each set of data?
V	Normality	Methods	13c	7b	When parametric statistical tests were used, did the analysis include tests for normality of the group data?
W	Homogeneity	Methods	13c	7b	When parametric statistical tests were used, did the analysis include tests for homogeneity of the group variances?
X	Design	Methods			Were the statistics used appropriately for the design of the experimental protocols?
Y	Euthanasia	Methods		16a	How were animals euthanized for experimental purposes or at the end of experiments?
Z	Baseline	Results	14		Were baseline characteristics (e.g., weight and heart rate) of subjects prior to experimental treatments reported?
AA	Animal numbers	Results	15a	3c	Was the number of subjects used for each group for each analysis reported?
BB	Vague numbers	Results	15a	3c	Was the number of subjects used for each group reported as a range of values?
CC	Figures/tables	Results		3c	Did the legends of figures and tables report both the numbers of subjects and the statistical tests used for each analysis?
DD	Attrition	Results	15b	3a,b	Did the paper report subjects or data that were excluded from the analysis?
EE	Distribution	Results		10a	Were estimates of the group distributions (mean, median, standard deviation, etc.) used appropriately?
FF	Precision	Results	16	10b	When appropriate, were the results of statistical tests reported with measures of precision (i.e., SE or CI)?
GG	Specific P	Results			Were specific P-values reported for statistical tests?
HH	Adverse events	Results	17	16b	Did the paper report adverse events that might have resulted from experimental treatments?
II	Bias	Discussion	18b	17b	Did the discussion address any potential sources of bias in the development, conduct, or analysis of experiments?
JJ	Limitations	Discussion	18b	17b	Did the discussion address the limitations of the experimental model and possible sources of imprecision of the results?
KK	3 Rs	Discussion	18c		Did any aspect of the report address the replacement, refinement, or reduction of the use of animals in the research?
LL	Generalization	Discussion	19	18	Did the discussion address ways that the findings might extrapolate to other species or human biology?
MM	Funding	Discussion	20	21b	Did the paper list funding sources and describe the role of the funding agencies?
NN	Conflicts			21a	Were conflicts of interest addressed?

With the exception of two modeling studies, we initially examined all articles in AJP:HC volume 312 (2017). Because of changes in the American Physiological Society (APS) publication guidelines in 2016, we decided to delay publication of those results and also examine a later volume of AJP:HC (volume 316, 2019) to assess whether those recommendations had a noticeable effect on the quality of the articles in AJP:HC. In addition, at that later time, we also used the same criteria for articles in Circ Research (volume 124) and CV Research (volume 115). In this later analysis, a planned pattern of selection was used; we examined the first eight articles of each month for AJP:HC, the first four to five articles for each month for CV Research, and three to seven articles for each month of Circ Research. Modeling articles were excluded from the analyses. As a result of this plan, the number of articles that were examined for each of these journals was 95 articles for AJP:HC volume 312 (2017), 48 articles for AJP:HC volume 316 (2019), and 30 articles each for Circ Research volume 124 (2019) and CV Research volume 115 (2019).

The questions that are associated with the categories in Table 3 were used to determine the adequacy of descriptions or information that was reported in the articles (herein, reference to journal articles also includes accompanying supplements). Each article that was selected was read carefully and evaluated independently by two reviewers. In general, each reader evaluated each project where animals were used on the categories in Table 3 with a 0-2 scale (not present, weak, and adequate, respectively). A single reviewer with statistical expertise (JLW) evaluated the design (category X). Many of the same questions were used for the assessment of human studies, except that categories that did not apply to humans were disregarded (categories D, M, N, KK, and LL). At this stage of the review, each rater was blinded to the other rater’s assessment. The inter-rater reliability (percent agreement) for assessment of adequacy was 93%. After this individual rating, a consensus score in each category for each paper was determined in a subsequent discussion between the two reviewers.

Circ Research was the only journal that we analyzed that included a required checklist for preclinical animal studies as a supplement with the published articles. For our analysis, these checklists were used for additional assessments of compliance and rigor; in this case, the categories used for our analysis (Table 3) were matched with the Circ Research checklists for common elements. For compliance, our guideline scores in several categories were used to assess whether the information was present or not as indicated by the authors in the checklist. In our analysis, overall compliance was equal to the percentage of articles that scored either 2 (adequate) or 1 (weak); i.e., 100% minus the percentage that scored 0 (i.e., absent). For assessment of rigor, the average scores (mean ± SD) and the percentages of reports that were adequate, weak, or absent were examined in these same categories.

Statistical analysis

In this study, our primary goal was to survey recent cardiovascular literature to determine whether the research met adequate standards for reproducibility. In considerations of power, preliminary results indicated that the performance of the published research would fall far short of acceptable criteria (i.e., power ≥ 0.80) for all the journals that we examined. Therefore, we did not perform inferential statistical comparisons between the journals in the analysis of power. In the assessment of adherence to guidelines, both descriptive and inferential statistics were used. Descriptive statistics were calculated with Microsoft Excel, version 16.49 (Microsoft Corporation, Redmond, WA). Inferential statistical analysis was performed with GraphPad Prism, version 9.1.0 (GraphPad Software, Inc., San Diego, CA).

Power

In the examination of the power capabilities of each study, all of the measurements (i.e., 20% and 50% change from the initial value) for each study were first summarized by determining the median value of the measurements. The number of measurements per study varied from 1 to 50, which was the upper limit that we used in the event that numerous unpaired t-tests were used in a study. Subsequently, of these median values for each study, the median, minimum, and maximum values, and 25th and 75th percentiles were determined. Power was determined with G*Power or R statistical software.

Guidelines

The primary outcome of this portion of the study was to survey journal articles and examine their adherence to the guidelines. For this assessment, descriptive statistics were used (i.e., mean, SD, median, and quartiles). For each of the 40 categories that were assessed for the research studies, the percent of the articles that were rated as adequate (score = 2) was determined.

A secondary goal of this study was to compare the raw adequacy scores (0-2) between the journals for each category (Table 3) with a Kruskal-Wallis test (Table 4) [15]. When the result of this test was significant (P < 0.05), post hoc analysis was performed by pairwise comparison with the Mann-Whitney U test with Bonferroni correction (overall P < 0.05). Analysis of variance was used to determine whether the median adequacy (%) of the 40 categories for each journal was similar in the journals. Because percentages form a binomial distribution rather than a normal distribution, the values were first modified with an arcsine transformation (f): f = 2 x arcsin x (square root of P), where P is the value of the percentage, which satisfies the assumption of normality for parametric statistics [15].

**Table 4:** Comparison of scores among journal categories. The descriptor categories are from Table 3. The P-value for each category resulted from the Kruskal-Wallis test. Post hoc significant differences between individual journals are indicated by letters (P < 0.05 with Bonferroni method): (a) AJP:HC 2017 vs. AJP:HC 2019; (b) AJP:HC 2017 vs. Circ Research; (c) AJP:HC 2017 vs. CV Research; (d) AJP:HC 2019 vs. Circ Research; (e) AJP:HC 2019 vs. CV Research; (f) Circ Research vs. CV Research. ^1 ^If no superscript is present by a descriptor, the analysis included both nonhuman and human subjects: numbers of articles were 95, 48, 30, and 30 for AJP:HC 2017, AJP:HC 2019, Circ Research 2019, and CV Research 2019, respectively. ^2 ^Analysis excluded articles with human subjects: numbers of articles were 74, 38, 26, and 28 for AJP:HC 2017, AJP:HC 2019, Circ Research 2019, and CV Research 2019, respectively. ^3 ^Power value was calculated as a single difference of 20% or 50% from an initial value from AJP:HC 2017 in three of the groups. For categories T and II in AJP:HC 2017, all individual values were zero; therefore, prospective power could not be calculated. * The overall P-value with the Kruskal-Wallis test was statistically significant, but after the Bonferroni adjustment, the calculated P-value for this comparison (.0128) was slightly short of the statistical cutoff of 0.008. AJP:HC = American Journal of Physiology: Heart and Circulatory Physiology; Circ Research = Circulation Research; CV Research = Cardiovascular Research.

	Descriptor^1^	P-value	Post hoc	Power^3^
A	Title	.278		.92/1.00
B	Abstract	.013	d	.79/1.00
C	Background	.165		.96/1.00
D	Model^2^	.019	b	.54/1.00
E	Objectives	.123		.88/1.00
F	IACUC/IRB	.070		1.00/1.00
G	Groups (IV)	.624		.67/1.00
H	Randomization	<0.0001	b, d	.07/.19
I	Blinding	<0.0001	b, d, f	.07/.15
J	Units	.762		1.00/1.00
K	How/why	.895		.96/1.00
L	When/where	<0.001	b, c	.07/.18
M	Animals^2^	.005	a	.59/1.00
N	Housing^2^	<0.001	d	.09/.36
O	Group numbers	.344		.13/.60
P	Power	<0.0001	b, d, f	.05/.08
Q	Allocation	.661		.17/.80
R	Treatment order	.109		.05/.07
S	Outcomes	<0.001	a	.99/1.00
T	1°/2° outcomes	.040	b*	—
U	Statistics	.440		.68/1.00
V	Normality	<0.0001	b, d, f	.07/.16
W	Homogeneity	.560		.05/.08
X	Design	.259		.58/1.00
Y	Euthanasia	.006	c, f	.15/.73
Z	Baseline	.004	a, e	.10/.40
AA	Subject numbers	.149		.72/1.00
BB	Vague numbers	.716		.08/.34
CC	Figures/tables	<0.0001	c, e	.34/.90
DD	Attrition	<0.0001	b, d, f	.06/.16
EE	Distribution	.320		.60/1.00
FF	Precision	.771		.41/1.00
GG	Specific P	.010	e	.09/.34
HH	Adverse events	<0.001	b	.05/.07
II	Bias	.095		—
JJ	Limitations	.138		.49/1.00
KK	3 Rs^2^	.282		.05/.06
LL	Generalization^2^	.104		.39/1.00
MM	Funding	.383		.99/1.00
NN	Conflicts	.768		1.00/1.00

For each of the 40 categories that were tested with a Kruskal-Wallis test, a power analysis calculation was performed (Table 4). For this a priori power calculation, the initial group values that were used for each category were the mean and SD from AJP:HC 2017. The calculation used this initial value in three groups and a difference of 20% in the fourth group. Because the scale used was ordinal and limited from 0 to 2, a 20% difference from each mean was calculated towards the center of the range. Therefore, if the mean was less than 1.00, a 20% increase was used; if the mean was equal to or greater than 1.00, a 20% decrease was used. The power analysis for each of these comparisons was done via a one-way ANOVA with G*Power. This substitution can be made because the power of the Kruskal-Wallis test is similar to that of a one-way ANOVA when the distribution is normal; when the distribution is skewed, the Kruskal-Wallis test is more powerful than the one-way ANOVA [16].

## Results

For ease of interpretation, the data presented in the results and discussion sections indicate the individual journals and years that the volumes were published; e.g., CV Research 2020 refers to volume 116 of CV Research, which included the issues that were published in January through June of 2020 (Table 1). Because the months of AJP:HC volumes 319 and 320 are continuous (July through December 2020 and January through March 2021, respectively), those datasets were combined for analysis and presentation.

Analysis of power

**Figure 1:**
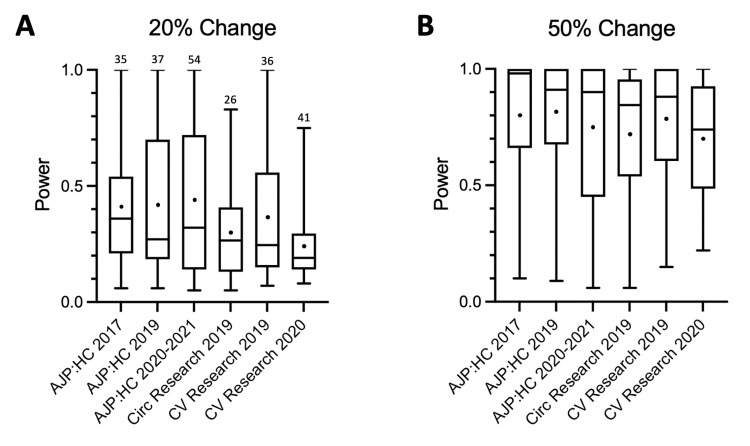
Power of unpaired t-tests for a 20% (A) and 50% (B) change from the initial values. The central bars in these box-and-whisker plots are the median values (50th percentile). The lower and upper limits of the rectangles represent the 25th and 75th percentiles, respectively. The lower and upper limits of the bars represent the minimum and maximum values, respectively (i.e., the range). The numbers over the maximum values in A indicate *n* for each journal for both A and B. AJP:HC = American Journal of Physiology: Heart and Circulatory Physiology; Circ Research = Circulation Research; CV Research = Cardiovascular Research.

The number of unpaired t*-*tests that was measured in each article varied from one to 50, with an average number per article of 12 for AJP:HC and 10 each for Circ Research and CV Research (Table 2). In this study, we examined the power of unpaired t*-*tests with a change of either 20% or 50% from the initial value. For a 20% change from the initial values (Figure 1A), the median power values for the journals examined varied from 0.19 to 0.36, with an average value of 0.27 ± 0.06 (mean ± SD). Therefore, the overall probability of a type II error for these t*-*tests was 0.73 ± 0.06. As expected, the power for a 50% change from the initial values was much greater than for a 20% change. For a 50% difference (Figure 1B), the median power values varied from 0.74 to 0.98, with an average median value of 0.88 ± 0.08. The type II error rate for these unpaired t*-*tests, therefore, was 0.12 ± 0.08. The median ES (*d*) for the unpaired t-tests in each journal volume varied from 0.68 to 1.00 (0.88 ± 0.11) for a 20% difference from the initial values and from 1.71 to 2.50 (2.21 ± 0.28) for a 50% difference.

**Figure 2:**
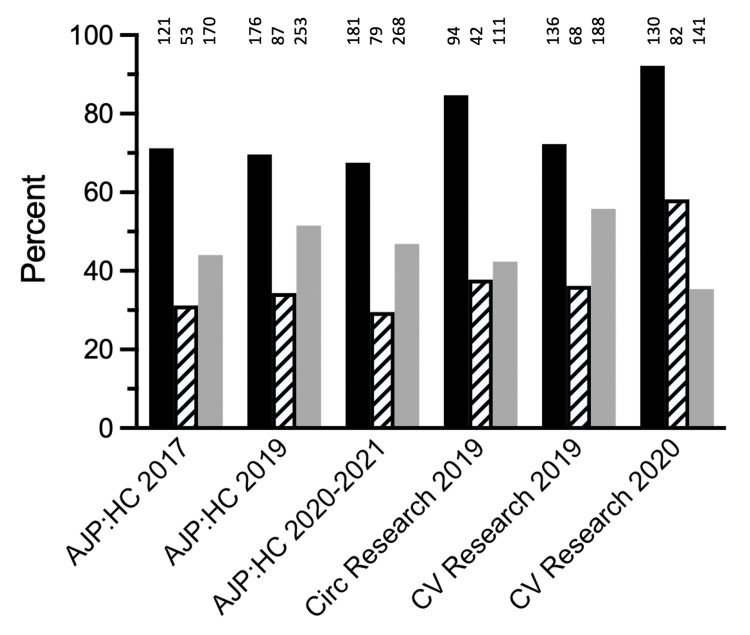
Percent of NS unpaired t-tests that did not achieve adequate power. The percent of NS unpaired t*-*tests that did not achieve the power of at least 0.80 for a difference of 20% or 50% from the initial values are shown by black and striped bars, respectively. The gray bars indicate the percent of the total tests analyzed for each journal volume that was NS. The numbers above bars indicate *n*. NS = not significant; AJP:HC = American Journal of Physiology: Heart and Circulatory Physiology; Circ Research = Circulation Research; CV Research = Cardiovascular Research.

An average of 46.0 ± 7.2% of the analyzed unpaired t-tests were reportedly not statistically significant (NS) in the six volumes sets that were analyzed (Figure 2). A high percentage of these NS tests had a power of less than 0.80. In the six volumes sets, for a change of 20% from the initial value, 76.3 ± 9.9% (range, 67.5%-92.2%) of the NS tests did not have power that equaled or exceeded 0.80. For a change of 50%, 37.9 ± 10.4% (range, 29.5%-58.2%) of the NS tests had power less than 0.80. For AJP:HC, the proportion of NS tests with power less than 0.80 ranged from 67.5% to 71.2% for a 20% difference from the initial value and 29.5% to 34.4% for a 50% difference. For Circ Research and CV Research, the values ranged from 72.3% to 92.2% for a change of 20% from the initial values and from 36.2% to 58.2% for a difference of 50%.

Analysis of guidelines

As with the ARRIVE format, the questions that were used for the examination of each paper (Tables 3, 4) were grouped in a manner that is similar to the layout of a research paper (title, abstract, introduction, methods, results, and discussion). The primary outcome for this portion of the study was to assess the compliance of each journal with the guidelines. Secondary outcomes included comparisons of adequacy scores between journals and examination of adherence to a checklist that was used by Circ Research 2019.

Title, Abstract, and Introduction

**Figure 3:**
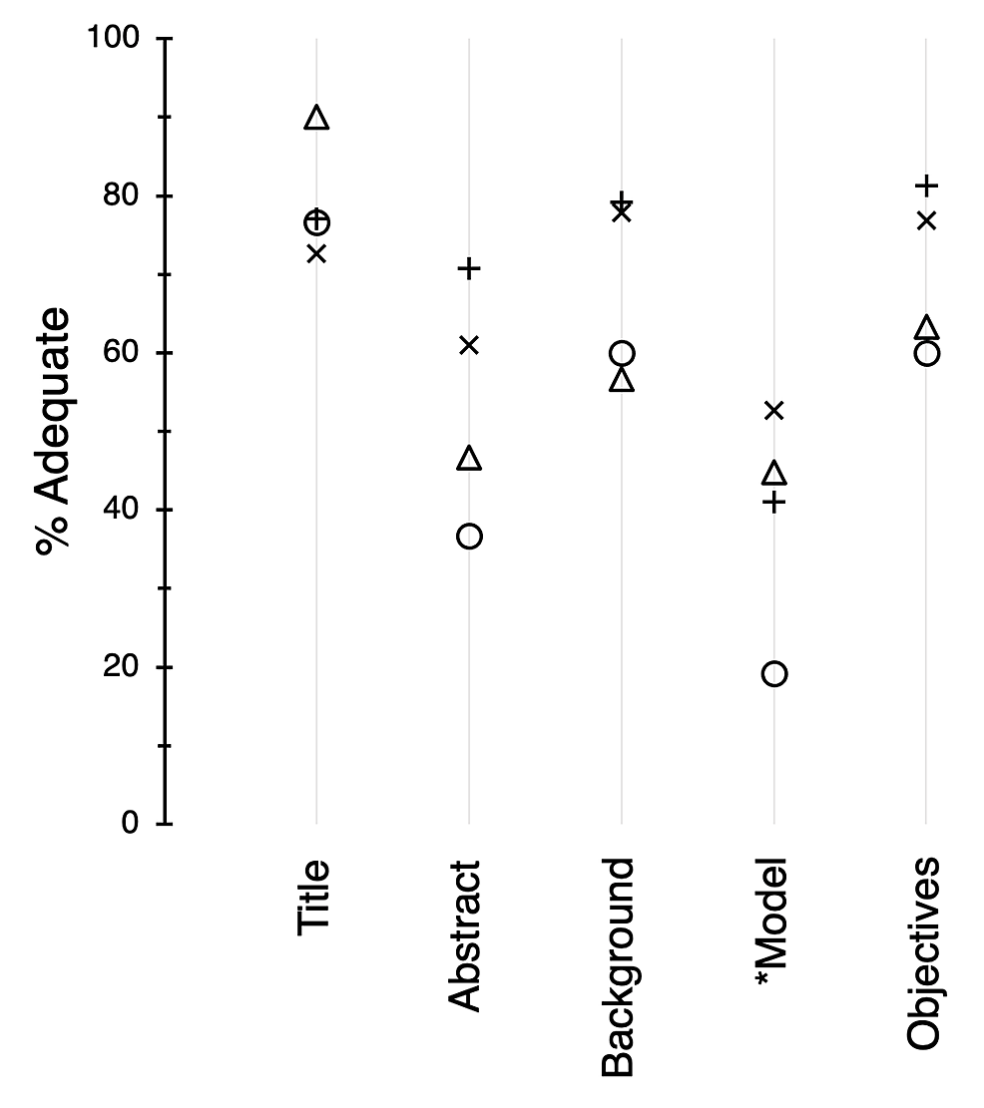
Adequacy scores (%) for categories of the title, abstract, and introduction. Symbols: X, AJP:HC 2017; +, AJP:HC 2019; O, Circ Research 2019; △, CV Research 2019. * Analysis excluded articles with human subjects: numbers of articles were 74, 38, 26, and 28 for AJP:HC 2017, AJP:HC 2019, Circ Research 2019, and CV Research 2019, respectively. Otherwise, all subjects were included: numbers of articles were 95, 48, 30, and 30 for AJP:HC 2017, AJP:HC 2019, Circ Research 2019, and CV Research 2019, respectively. AJP:HC = American Journal of Physiology: Heart and Circulatory Physiology; Circ Research = Circulation Research; CV Research = Cardiovascular Research.

Overall, the titles of the journal articles (Table 3, category A) conveyed enough meaningful information to give readers some indication about the nature of the studies. The mean adequacy (% adequate) among the journals for the title was 79% (Figure 3). The abstracts were examined for the overall content, including sufficient summaries of the background, objectives, methods, results, and conclusions (category B). For abstracts, adequacy varied considerably among journals (P = 0.013), with scores ranging from 37% for Circ Research to 71% for AJP:HC 2019.

According to the ARRIVE guidelines (Table 3), the introduction should include relevant background information, the rationale for the study, the experimental approach and its appropriateness, and statements that describe the objectives and hypothesis. For the introduction, adequacy scores for the background (C) and objectives (E) were moderately high, with mean scores of 68% and 70%, respectively. Scores for the experimental approach (D) were lower and variable (range, 19%-53%; P = 0.019).

Methods

**Figure 4:**
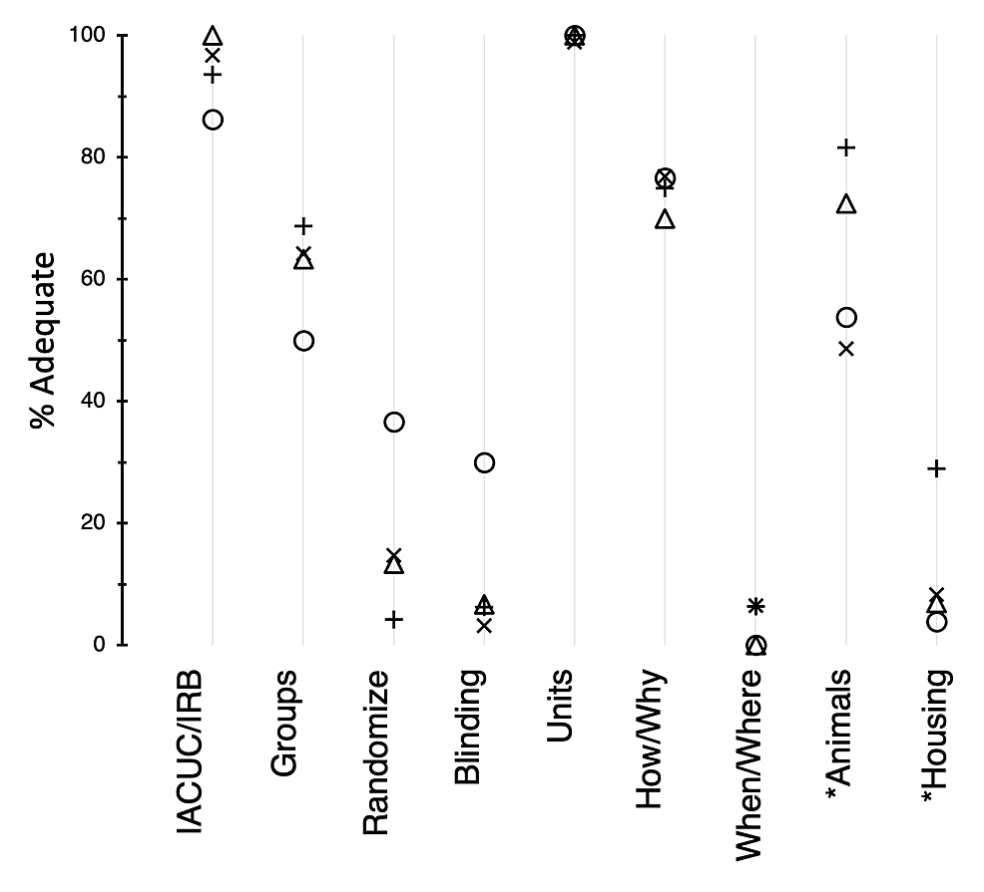
Adequacy scores (%) for categories of the methods section. Symbols: X, AJP:HC 2017; +, AJP:HC 2019; O, Circ Research 2019; △, CV Research 2019. For *n*, see the legend of Figure 3. * Analysis excluded articles with human subjects. AJP:HC = American Journal of Physiology: Heart and Circulatory Physiology; Circ Research = Circulation Research; CV Research = Cardiovascular Research.

For the methods section (Figure 4), the guidelines indicate that journal articles should describe a process for ethical review (F), and the articles generally scored high in this category (mean, 94%). The scores for the description of the groups in the methods section (G) were moderate (mean, 62%). Reporting of the use of randomization (H) and blinding (I) was moderately low for Circ Research and extremely low for the other three journals (P < 0.0001). For Circ Research, randomization and blinding adequacy scores were 37% and 30%, respectively. For the other journals, mean scores were 11% and 5%, respectively. In contrast, it was almost always apparent what units were measured (J) in the research.

In the methods section (Figure 4), the ARRIVE guidelines recommend that research articles describe precisely how things were done and what the rationale was for these methods (K). With an average score of 75% in this category, the performance was relatively strong. However, reporting of the precise details about the time and place of the experiments (L) was extremely weak (mean, 3%). For animal studies, the scores for descriptions of the nature and sources of animals that were used in the experiments (M) were moderately high (mean, 64%). The reporting of animal housing and husbandry conditions (N) was variable (P < 0.001) and clearly inadequate (range, 4%-30%).

**Figure 5:**
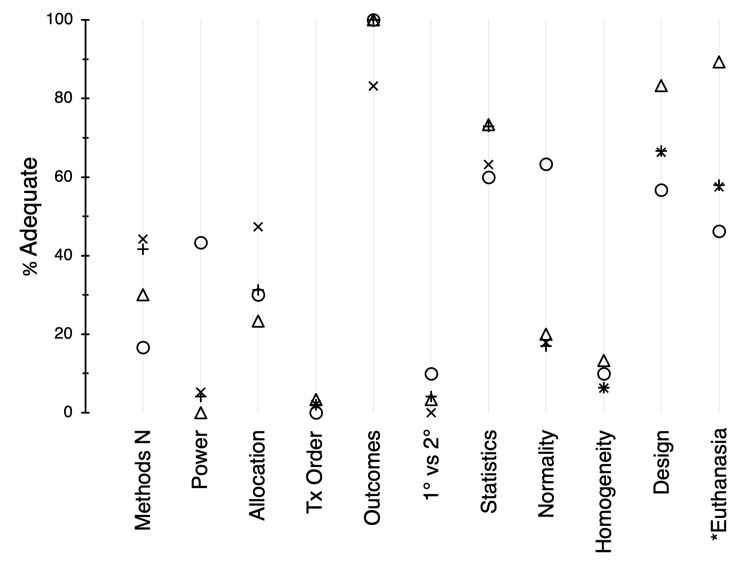
Adequacy scores (%) for categories of the methods section (continued from Figure 4). Symbols: X, AJP:HC 2017; +, AJP:HC 2019; O, Circ Research 2019; △, CV Research 2019. For *n*, see the legend of Figure 3. * Analysis excluded articles with human subjects. AJP:HC = American Journal of Physiology: Heart and Circulatory Physiology; Circ Research = Circulation Research; CV Research = Cardiovascular Research.

The ARRIVE guidelines recommend that the overall number of animals used and the numbers for each experimental group be described clearly in the methods section of a research paper (Figure 5; category O). The adequacy scores were low in this category (range, 17%-44%). With the exception of Circ Research (43%; P < 0.0001), a power calculation or other explanation of how the group sizes of experimental subjects were determined (category P) was usually missing. Adequacy scores for the descriptions of how subjects were allocated to treatment groups (Q) ranged from 27% to 47% (P = 0.661). Information on the order of the treatment or assessment of subjects within each group (R) was almost always absent (mean, 2%). In contrast, the nature of the experimental outcomes of interest (S) was usually very clear in these papers (range, 83%-100%). However, differentiation of primary and secondary outcomes (T) was very weak (mean, 3%).

In the majority of articles (Figure 5), the statistics (category U) were described adequately in the methods section (mean, 67%). Assumptions for parametric data include normality (V) and homogeneity of variance (W); with the exception of Circ Research, tests for normality of parametric data were usually not reported (Circ Research, 63%; mean for other journals, 18%). Tests for homogeneity of variance were generally absent (mean, 9%). Overall, the selection of statistical tests (X) was appropriate for the experimental design (range, 57%-83%). With the exception of CV Research, for animal studies, an average of 54% of research articles contained an adequate description of euthanasia procedures (Y); no description of euthanasia methods was presented in 43% of these articles. In contrast, the adequacy score for CV Research for euthanasia methods was 89% (P = 0.006).

Results

**Figure 6:**
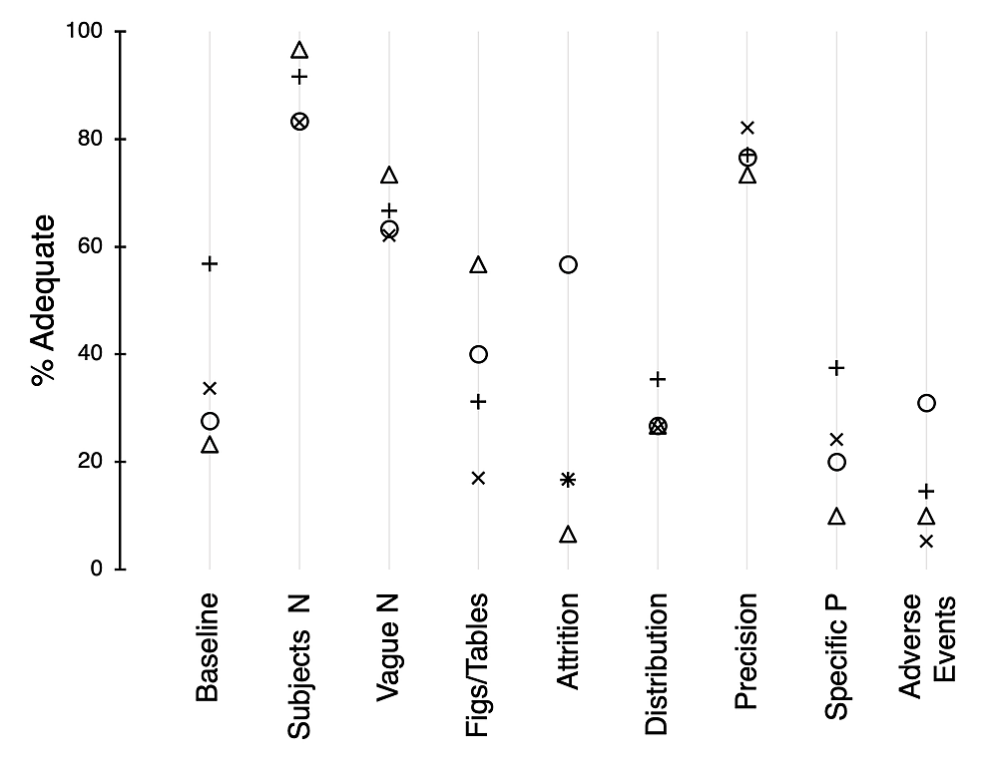
Adequacy scores (%) for categories of the results section. Symbols: X, AJP:HC 2017; +, AJP:HC 2019; O, Circ Research 2019; △, CV Research 2019. For *n*, see the legend of Figure 3. AJP:HC = American Journal of Physiology: Heart and Circulatory Physiology; Circ Research = Circulation Research; CV Research = Cardiovascular Research.

With the exception of AJP:HC 2019 (adequacy, 57%; P = 0.004), the reporting of baseline characteristics of experimental subjects (category Z) was relatively low (mean, 28.2; Figure 6). Reporting of the numbers of animals used (AA) was strong in the results section (mean, 89%). In most cases (mean, 66%), specific numbers were used in the figures and tables rather than ranges of values (BB), which aided readers in the interpretation of results. The guidelines recommend that each of the figures and tables contains information on the specific numbers and statistics that are used (CC). In this category, the adequacy scores were variable (P < 0.0001; range, 17%-57%). Reporting of subject attrition (DD) throughout the studies varied (P < 0.0001), with the majority of the articles in Circ Research describing the loss of subjects (57%); in contrast, reporting of these exclusions in the other journals was scanty (mean, 13%).

Guidelines suggest that indices of both distributions (i.e., mean ± SD or median and quartiles; EE) and precision (i.e., confidence interval or standard error; FF) be used and reported in research in appropriate circumstances (Figure 6). Whereas adequacy scores for distribution were moderately low (mean, 29%), those for precision were relatively high (mean, 77%). Use of specific P-values when reporting the results of statistical tests (GG) was variable and low (P = 0.010; range, 10%-38%). Similarly, reporting of adverse events (HH) was also variable and low (P < 0.001; range, 5%-31%).

Discussion and Acknowledgements

**Figure 7:**
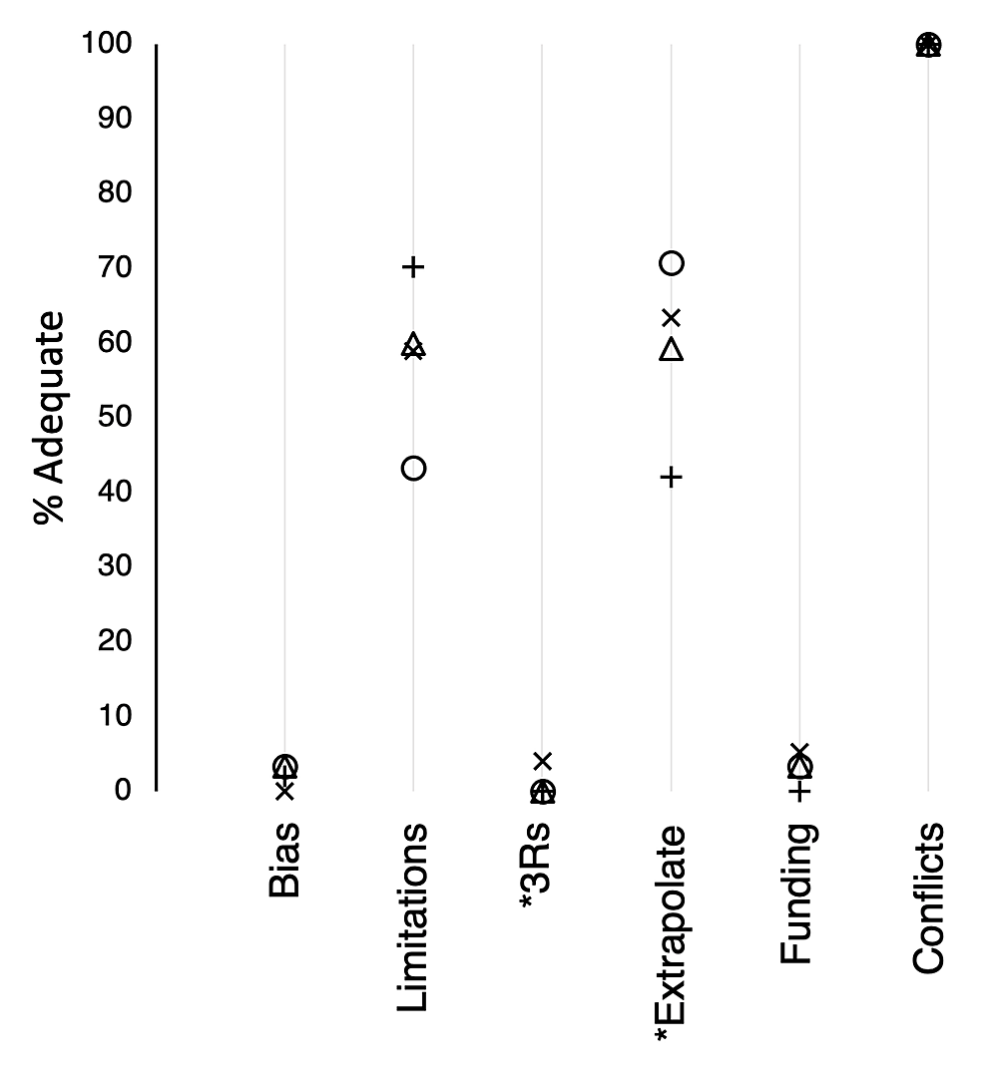
Adequacy scores (%) for categories of the discussion and acknowledgements sections. Symbols: X, AJP:HC 2017; +, AJP:HC 2019; O, Circ Research 2019; △, CV Research 2019. For *n*, see the legend of Figure 3. * Analysis excluded articles with human subjects. AJP:HC = American Journal of Physiology: Heart and Circulatory Physiology; Circ Research = Circulation Research; CV Research = Cardiovascular Research.

In these research articles, discussion of potential biases (category II) was almost nonexistent (mean adequacy, 3%; Figure 7). Discussion of the limitations of the model and experiments (JJ) was adequate in the majority of research reports (mean, 58%). Possibilities for replacement, refinement, or reduction of the use of animals (KK) were only addressed in three papers. For animal studies, generalization of results to other species or humans (LL) was adequately discussed in an average of 59% of the research reports. Whereas all of the articles that had funding listed their funding sources (MM), very few of these (mean, 3%) mentioned the role of the funder, as recommended by the guidelines. In contrast, all of the articles addressed potential conflicts of interest (NN) adequately.

Comparison of Overall Adequacy Scores

The median value for percent adequacy of all 40 categories in each journal volume was 47.4% for AJP:HC 2017, 41.9% for AJP:HC 2019, 43.3% for Circ Research, and 45.8% for CV Research. The median values were similar for the journals (P = 0.973; power for a 20% change = 0.69).

Adherence to Checklist

For Circ Research, the required checklists that were attached as supplements [17] with the research articles were useful for additional analysis of compliance and robustness (Table 5). Several of the categories in the Circ Research checklist overlapped with the categories that we used for analysis in the present study (Table 3). In some of these categories (Table 3: H, I, P, T, V, DD, and HH), although the percent adequacy for Circ Research was moderately low, the scores were significantly elevated compared to some of the other journals (Table 4). Visual inspection of Figures 5-7 highlights the elevations in Circ Research for randomization, blinding, power, normality, attrition, and adverse events. This observation suggests that the use of a checklist by Circ Research increased the adequacy in these categories.

**Table 5:** Categories from Table 3 that overlap with those of the checklist in Circ Research 2019 (n = 26). Refer to Table 3 and Circ Research checklist [17] for more detailed information on the categories. ^† ^Significantly greater adequacy scores (P < 0.05) for Circ Res 2019 (Table 4). Categories in Circ Research checklist: ^1 ^study design, ^2 ^randomization, ^3 ^blinding, ^4 ^sample size and power calculations, ^5 ^data reporting, ^6 ^statistical methods, and ^7 ^experimental details, ethics, and funding statements. IACUC = Institutional Animal Care and Use Committee; IRB = Institutional Review Board; Circ Research = Circulation Research.

Category	Descriptor	Score (mean ± SD)	Adequate (%)	Weak (%)	Absent (%)	Mismatch (%)
F	IACUC/IRB^7^	1.86 ± 0.35	86	14	0	0
G	Groups^1^	1.50 ± 0.51	50	50	0	0
H^†^	Randomization^2^	1.07 ± 0.83	37	33	30	3
I^ †^	Blinding^3^	1.17 ± 0.65	30	57	15	3
M	Animals^5^	1.54 ± 0.51	54	46	0	0
N	Housing^5^	0.19 ± 0.49	4	11	85	77
P^ †^	Power^4^	1.00 ± 0.95	43	13	43	23
T^ †^	1°/2° outcomes^1^	0.17 ± 0.53	7	3	90	50
U	Statistics^6^	1.57 ± 0.57	60	37	3	0
V^ †^	Normality^6^	1.43 ± 0.82	63	17	20	10
AA	Animal numbers^1^	1.83 ± 0.38	83	17	0	0
DD^†^	Attrition^5^	1.20 ± 0.96	57	7	37	17
EE	Distribution^6^	1.20 ± 0.55	27	67	7	3
FF	Precision^6^	1.57 ± 0.82	77	3	20	13
HH^ † ^	Adverse events^5^	0.69 ± 0.93	31	7	62	14
MM	Funding^7^	1.03 ± 0.18	3	97	0	0
NN	Conflicts^7^	2.00 ± 0.00	100	0	0	0

Overall compliance with the checklist, as indicated by the percentage of articles that received a score of either 2 or 1 in a category, was relatively strong for reporting for Institutional Animal Care and Use Committee (IACUC) (F), groups (G), blinding (I), animals (M), statistics (U), normality (V), animal numbers (AA), precision (FF), funding (MM), and conflicts (NN). However, in some of these categories (G, I, M, U, and MM), many of the reports were judged to be weak and, therefore, inadequate.

In some categories, items were indicated to be present on the Circ Research checklist [17], but the information was absent (i.e., score of 0) in the article and supplement (Table 5). This inconsistency was most obvious for randomization (H), housing conditions (N), power calculations (P), identification of primary and secondary outcomes (T), attrition (DD), and adverse events (HH). In several categories, there was a mismatch in the use of the checklist and what was actually reported in the journal articles and supplements. For example, in 50% of the articles, the checklists indicated that the primary and secondary outcomes (T) were clarified, but the articles themselves did not mention or discuss this item. Large mismatches also occurred with information on housing conditions (N) and power calculations of sample size (P).

## Discussion

The reproducibility of research results is dependent on many factors. Currently, a major challenge for other investigators is the replication of the experimental methods and results that are reported in research journals. The primary objective of this study was to assess the quality and rigor of the research that has been published recently in prominent cardiovascular research journals by measuring statistical power and assessing compliance with accepted research guidelines. A secondary goal was to determine whether the use of a required checklist improved the compliance and quality of reporting.

Analysis of power

The findings of this study indicate that unpaired t*-*tests had the low statistical power to detect a reasonably large difference from the initial value (20%) in three different cardiovascular research journals. Power was unacceptably low in all of the journal volumes that we examined. Low power is widespread in clinical and preclinical research practices [18,19].

In the absence of other bias, statistical power is the probability that a positive statistical test from samples of a population can detect a difference that actually exists in the overall population. In addition, calculations of power are used to determine the probability of type II errors (i.e., false negatives), because the type II error rate (beta) equals 1 - power [12]. Therefore, with experiments of low power, the probability of a finding that is statistically nonsignificant being false in the population is high. In addition, the probability of a type I error (i.e., false positive) is also increased [19]. In situations of low power, because there are high rates of both false positives and false negatives, the positive and negative predictive values are both reduced. With low statistical power, therefore, the probability of research findings being reproducible for a population is decreased.

A probability value of 0.80 has become the standard as the minimally acceptable level for statistical power [12], and calculations are based on N, the cutoff P-value (alpha), and ES, which is calculated from SD and the difference between groups. In general, Cohen laid out values for different tests that are often used as reference points [12]. For the unpaired or independent samples t*-*test, ES, sometimes called “Cohen’s d,” is calculated as the difference between the two means divided by SD. Low, medium, and high minimum ES values are 0.2, 0.5, and 0.8, respectively. However, because Cohen’s work pertained to psychology, these standards may not apply well as markers of ES for some other fields. In this study, which examined biomedical research with a diverse array of types of subjects and experimental settings, we used a 20% or 50% difference from the initial value as ES. Our findings demonstrated a calculated ES of 0.88 for a 20% difference and 2.21 for a 50% difference; both of these values exceed Cohen’s values for high ES for the unpaired t-test.

In practice, an important use of power calculations is the prospective determination of the group size that is needed before the experiments begin [12]. These a priori calculations use alpha (usually 0.05), the minimally acceptable power level (usually 0.80), and the estimated ES. The means and SD values that are needed for ES calculations are often estimated from the literature or previous or preliminary experiments. In practice, the difference between means that is needed for power calculation of group size for an unpaired t*-*test is the expected difference or that difference that is considered to be meaningful.

Two factors can be responsible for decreased power of measurements: a large standard deviation or a small group size. In rare cases, the standard deviation might be decreased through better experimental designs that reduce the variability between subjects or increase the precision of measurements. Most often, however, the most obvious solution is to increase the size of the experimental groups. Attempts to increase the size of groups can present a conundrum for investigators because large increases in subject numbers impact often limited financial resources and time of personnel. However, it can be argued that conducting experiments with extremely low power is fiscally irresponsible and a waste of resources and time because the results from those experiments may have high probabilities of type I and type II errors and be highly irreproducible [19].

One possible solution for increasing group size was proposed by Bonapersona et al., i.e., greatly increasing the size of control groups by using previous data from other studies that used similar controls [18]. Another approach for investigators is to more greatly differentiate between primary and secondary outcome variables than is done currently. In this case, the focus of the study is shown by the identification of primary outcomes, and secondary outcomes should be used to support the primary ones or to identify preliminary findings that are less reliable [20]. With too many outcome variables treated as primary, the focus of a study becomes confused. Prospective power analysis should only be done for the primary outcome variables, with group sizes conforming to those calculations. With this approach, other variables are considered to be secondary, and those findings are viewed as preliminary. For the secondary results, power values should be presented transparently so that readers can see the probabilities for type I and type II errors. In the present study, the identification of statistical differences between ARRIVE scores (Table 4) was a secondary objective. The power values for those comparisons are presented for the purpose of transparency.

Analysis of guidelines

In the cardiovascular research journals that we assessed, examination of the categories in an augmented ARRIVE checklist highlighted both strengths and weaknesses of the research reports, and our findings are consistent with previous surveys [5,7-11,21-23]. Categories of the greatest strength and consistency included the format of the title and some elements of the introduction; reporting to institutional ethics and approval committees; explanations and rationale for the units used; precision and the overall experimental and statistical design; and reporting of experimental outcomes and conflicts of interest. In the discussion section, coverage of experimental limitations and generalizations to other populations was moderately strong.

In contrast, other key elements were unacceptably weak. In three of the journal volumes, little mention was made of power calculations for determination of group size, elements of experimental design that reduce bias (i.e., allocation, randomization, and blinding), adjustment for non-normal frequency curves, and attrition and adverse events of experimental subjects. In many of the journal articles, differentiation between primary and secondary outcomes and the use of standard deviations and specific P-values was low. In the discussion sections, consideration of possible bias and “the 3Rs” (i.e., possibilities for the replacement, refinement, or reduction of animal use) [13] was almost nonexistent. Similarly, the role of funders in research projects was almost never mentioned.

ARRIVE and other guidelines that were developed for the reporting of preclinical research resulted from the increasing awareness that the ethics and reproducibility of much existing research were questionable [13]. During the last decade, the recognition of these issues has been apparent and widespread, resulting in over 1,000 journals supporting the use of the ARRIVE guidelines [6]. Despite this support, compliance by authors has been poor, and key elements that are needed for reproducibility continue to be neglected in laboratory practice or underreported in journal publications. Since the ARRIVE guidelines were first published in 2010, numerous surveys of compliance to research guidelines have reported either no improvement in research practices and reporting or only modest improvements that were still inadequate for reproducibility [5,7-10,22].

Among the most detrimental deficits that have been noted in this and other surveys is the low prevalence of power calculations for the determination of group size and procedures for the reduction of bias [7,9,10,21,23]. Low power and different types of bias can lead to effects that are falsely exaggerated. In research, bias refers to characteristics of a sample that are systematically different from those in a population. Situations where bias exceeds the true effect lead to false-negative results or falsely augmented or diminished findings.

Although bias can be introduced into research intentionally, it most often results from unintentional oversights in the methods and processes. Bias can occur at all levels of the experimental process, from the generation of a hypothesis and design of experiments to the publication of results. A variety of types of selection bias can result from nonrandom sampling, group assignments, and allocation order or discounting attrition of subjects during the course of experiments. Blinding at different times during treatment and data collection and analysis reduces the incidence of expectation bias, observer bias, and other types of measurement bias. Indeed, a study by Bello et al. indicated that the findings of nonblinded experiments were exaggerated by 59% [21]. Similarly, studies without randomization or blinding are more likely to show differences between groups than those without these protocols, presumably because of bias [24]. Publication bias is a major problem and can lead to exaggerated positive findings. The push by institutions and journals to publish unique findings with large statistically significant differences has resulted in the greatly reduced publication of negative studies, encouraged confirmation bias, stifled the replication of previous studies, and dampened exploratory research [3,25].

To increase the likelihood that research can be replicated, the ARRIVE guidelines were designed to encourage transparency in all aspects of reporting in a publication [13]. Therefore, ARRIVE categories were developed to detail the characteristics of the title, abstract, methods, results, and discussion of a research paper. In our study, we observed that the descriptions were incomplete in some sections (Figures 3-7). For example, in the introductions of the research reports, the background information and objectives were generally clear, but a methodological overview was often sketchy or absent. In the methods sections, the description and rationale for procedures were often strong, but critical details on the timing and locations of experiments were usually lacking. Whereas descriptions of the animals used in the experiments usually were adequate, information about the housing and husbandry conditions was almost always absent. Descriptions of the baseline health of animals, loss of animals, and adverse events during the course of the studies were usually weak or absent. In almost every case, discussions of potential bias were missing, and for animal studies, discussions of possibilities for the replacement, refinement, or reduction for animal use (i.e., “the 3Rs”) were absent. The ratings for funding were almost always rated as inadequate because the ARRIVE guidelines recommend that both the source of funding and the role of the funders be discussed. Usually, the source of funding was listed, but the role of the funder was not described.

Consistent with other studies [10,11,26], our findings suggest that the inclusion of a checklist improves the quality of the research reports. In this study, reporting of randomization, blinding, power calculations, differentiation between primary and secondary outcomes, normality of frequency curves, attrition, and adverse events was greatest for Circ Research, which required the use of a checklist [17]. However, even with the use of a checklist, compliance for use or reporting remained too low, a finding also consistent with previous studies [10,11]. In addition, our findings showed that a high percentage of articles abused the Circ Research checklist, indicating the inclusion of information on the checklist that was actually missing in the article or supplement (Table 5).

Study limitations

This study was designed to accommodate the temporal and financial restrictions of the research programs of six medical students (HC, MKL, JD, RDM, DP, and RI) who worked with their faculty sponsor (JLW) on this research project during summer breaks and other available times. To meet these constraints, a planned systematic pattern was used for the selection of the articles, which included the first articles from each month in a journal volume. In addition, some articles in Circ Research were excluded that required extra costs beyond the standard library subscription for our university. In our study of power, we only examined the power of unpaired t-tests because of its simplicity of measurement and widespread use. We recognize that the nonrandom nature of article selection and the study of power with only unpaired t-tests increase the possibility of bias and reduce the generalization of our findings. However, our findings in three prominent cardiovascular research journals complement a large and growing body of literature in medical research that demonstrates that many research studies may not be reproducible because of weaknesses in experimental design, implementation, or reporting.

Inherent in a survey study of this type is a degree of subjectivity. Even with detailed descriptions guiding the assessor of the analysis of the guidelines, sometimes discerning the difference between an adequate or weak presentation in a category was difficult. Therefore, the protocol for these assessments was designed to reduce subjective bias. Initially, each article was carefully reviewed and assessed independently by two different reviewers. Inter-rater reliability of 93% has assured us that the agreement from these individual assessments was high. Second, after the initial independent reviews, a discussion was held together with the two reviewers to finalize a consensus on the ratings. In one category, experimental design (category X in Table 3), a single reviewer was used because a high degree of statistical expertise was required for the assessments, and only one statistician (JLW) was available for these assessments.

The ARRIVE guidelines were first developed for preclinical in vivo research studies. However, they are also quite useful for in vitro and human studies because many of the elements of the guidelines are ubiquitous, including overall experimental design, reduction of bias, use of statistics, and the presentation of each section of a research paper that is needed for transparency and reproducibility. In addition, most preclinical in vitro studies in biomedical research involves animals at some stage of the research; therefore, descriptions of animals and animal husbandry conditions are usually also needed. For human studies, guideline categories that were specific for the use of animals were omitted from the analysis.

A major finding of this paper was the demonstration of the low and inadequate power that was present with statistical tests. Notably, the data in Table 4 also demonstrated a number of low power values for the statistical group comparisons. Properly used, before experiments begin, prospective power calculations for the determination of the group size should be used only for primary outcome variables [20]. Secondary outcomes may have low power and should be considered to be supportive of the primary outcomes examined or to be preliminary or exploratory in nature, perhaps serving as a springboard for future studies. For practical and financial reasons, a study should only focus on a few primary outcomes, and the power calculations that determine the size of the study are based around those variables. Our analyses of power and the guidelines were designed primarily to be descriptive surveys that focused collectively on the quality of experimental design and reporting in the journals. In this study, comparisons between the journals were considered to be secondary outcomes because of the existence of low power and adequacy scores in all the journals. Our intention has been to highlight the strengths and stark shortcomings that exist in all the journals that we examined, even in those cases where one journal’s performance was apparently different than the others. In addition, in this paper, we have modeled how power can be presented transparently so that the reader can more fully examine the statistical results with the full acknowledgment of possible type I and type II errors. Moreover, with more transparency, subsequent investigators better understand the issues and challenges that are related to reproducibility.

Reproducibility

Recent studies have demonstrated that there are numerous controllable factors that can greatly improve the likelihood of replication of preclinical research. Experimental irreproducibility of preclinical research usually can be attributed to weaknesses in study design, quality control, protocols, materials and reagents, data analysis, or reporting, and publication bias also appears to play a major role [2,3,27,28].

Clearly, much needs to be done to remediate the shortcomings of published preclinical research. However, the description of the current situation as a “reproducibility crisis” is an overstatement that demonstrates a lack of understanding of the true nature of preclinical research and its role in translational medicine. To many, the use of the term “reproducibility” means “identical”; however, replication of identical conditions from one study to the next is virtually impossible because undetectable differences, errors, or biases in the experimental conditions or human factors may lead to different results [29]. Even with complete, transparent reporting of research protocols and methodology, the likelihood of creating the exact conditions of an earlier study by a different laboratory is extremely low.

Perfect reproducibility is unrealistic because it is neither practical nor affordable. Even with good adherence to guidelines and checklists, there are no acceptable standards in use that describe the level of detail needed in research reports that would ensure perfect replication. In addition, a supposition that reproducibility can be exact belies the fact that measurements and analyses in a study are often associated with some degree of imprecision that decreases the probabilities for exact duplication [27]. In addition, most biomedical research is inferential and probabilistic; therefore, even with similar conditions, identical results may not occur [28]. A calculated P-value of less than 0.05 on a statistical test with samples does not necessarily prove a finding is true in the population at large because of chance selection or type I or type II errors. Moreover, some heterogeneity of the design and conduct of experiments increases the robustness and generalization of the findings [30]. Indeed, some investigators have suggested that such “deliberate heterogeneity” is desirable and should become a standard part of experimental design [28].

The word “reproducibility” has been used both narrowly and broadly, so there is considerable confusion about its meaning. Perhaps other terms, such as “replication” or “corroboration,” should be used because they better describe the actual nature of research studies that use similar conditions as earlier studies, with the understanding that exact reproduction of the findings of a study is not an expectation [4]. Because of its limitations, a single study is not in itself definitive proof of its findings. Rather, most preclinical research studies are exploratory and should be considered as an initial part of a discovery process. Bollen et al. [4] have described this role elegantly: “Scientific knowledge is cumulative. The production of each empirical finding should be viewed more as a promissory note than a final conclusion.” As a result, initial studies are more susceptible to false positives than later confirmatory studies [30]. Initial findings are a first installment in the process of discovery that should be followed by important later studies that can possibly confirm the findings in different laboratories and different settings [27,30]. Because of the nature of this discovery process and the uncertainties involved, failure to replicate identical results by studies that follow an original study does not necessarily negate the findings of the earlier study.

Recommendations

During the last dozen years, journals that publish peer-reviewed preclinical research have taken steps that hopefully would improve the quality and reproducibility of the published research. ARRIVE and some other meaningful guidelines have been widely endorsed. Many journals have increased transparency and experimental details by publishing supplemental material and some have adopted checklists. The guidelines used by researchers and journals should be comprehensive, and they should eventually be used in their entirety by researchers when reporting a research project. The ARRIVE guidelines are well suited for this purpose because their categories are designed to inform readers in a clear and transparent manner at every level of a research publication, starting with the title and abstract, then continuing through the introduction, methods, results, and discussion sections [14]. The guidelines themselves can be used as a tool for determining the completeness and rigor of a research study and its published report by both researchers and journal reviewers and editors. The recently updated ARRIVE guidelines [14] have separated 21 categories into two sets: the Essential 10 and the Recommended Set of 11 categories. The Essential 10 represents a higher prioritization for the elements of a report that are considered to have the greatest influence on the replicability of a study, with the understanding that the greatest probability for reproducibility comes with adherence to the complete set of ARRIVE guidelines.

In general, the adoption of guidelines by research journals has not yet improved their quality and robustness to an adequate level [8-10]. At the present time, noncompliance with the guidelines that are recommended or required by research journals is a major problem [22]. For example, our results from the Circ Research checklist suggest that compliance is often poor, even when researchers are required to certify actions or inclusion of items in a research publication. These observations indicate that greater oversight is needed by reviewers, editors, and publishers. Perhaps each article should be reviewed for compliance by a separate reviewer who is trained in the guidelines rather than by the content experts who are usually called upon to review the scientific merits of a submitted manuscript. As we have demonstrated with this study, almost all of the categories in guidelines that are used by journals can be validated by a trained scientist without specific content expertise. In addition, the guidelines and checklists that are used by some journals currently need reexamination and strengthening for completeness.

Perhaps the greatest overarching challenge for reproducibility is to change the culture for research as it exists today, which is a carryover from decades of traditional approaches for the implementation and support of research programs. Many of these factors served well in earlier times, but this research culture has not changed enough to satisfactorily address today’s scientific, economic, and political challenges and realities. This entrenched traditional culture continues to promote attitudes and activities that greatly promote publication bias, which contributes to the failures in the reproducibility of published research. Academic institutions, publishers, and funding agencies continue to promote a publish-or-perish environment that encourages publication of initial positive findings when confirmatory or studies with negative results are frequently ignored. Although improving, weak transparency within publications or between researchers has traditionally led to secrecy, when greater collaboration and transparency are needed to increase the reproducibility and generalization of research findings. The greatest improvements in reproducibility will come as a result of top-down changes in the scientific culture that is led by academic institutions, funding agencies, and publications.

## Conclusions

The findings of the present study highlight an issue for cardiovascular research that has become apparent throughout most areas of biomedical research - the likelihood for reproducibility and translation of research findings to a large extent is low due to avoidable errors and oversights in the design, conduct, and reporting of the experiments that are published. In this study, while some areas of the examined reports were reasonably strong, key areas were not reported adequately or were omitted altogether. Many of the examined studies were underpowered and had a large probability for unintentional bias. It was difficult to discern whether the failings in the research occurred in laboratory or clinical settings or were primarily shortcomings in reporting of the published articles.
